# Bound to Lose: Physical Incapacitation Increases the Conceptualized Size of an Antagonist in Men

**DOI:** 10.1371/journal.pone.0071306

**Published:** 2013-08-07

**Authors:** Daniel M. T. Fessler, Colin Holbrook

**Affiliations:** Department of Anthropology and Center for Behavior, Evolution, and Culture, University of California Los Angeles, Los Angeles, California, United States of America; University of Stirling, United Kingdom

## Abstract

Because decision-making in situations of potential conflict hinges on assessing many features of the self and the foe, this process can be facilitated by summarizing diverse attributes in a single heuristic representation. Physical size and strength are evolutionarily ancient determinants of victory in conflict, and their relevance is reinforced during development. Accordingly, size and muscularity constitute ready dimensions for a summary representation of relative formidability, a perspective paralleled by the notion that social power is represented using envisioned relative size. Physical incapacitation constitutes a significant tactical disadvantage, hence temporary incapacitation should increase the envisioned size and strength of an antagonist. In Study 1, being bound to a chair increased men’s estimates of the size of an angry man and decreased estimates of their own height. Study 2 conceptually replicated these effects: among men for whom standing on a balance board was challenging, the attendant experience of postural instability increased estimates of an angry man’s size and muscularity, with similar patterns occurring at a reduced level among all but those whose equilibrium was apparently unaffected by this task.

## Introduction

Fight, flee, or parley? Successfully navigating antagonistic social interactions hinges on assessing one’s strategic assets and liabilities relative to those of one’s opponent. Importantly, physical capabilities are an elementary consideration in this regard. Here, we explore how temporary physical handicaps affect able-bodied men’s conceptualizations of prospective adversaries.

To determine the optimal course of action in situations of potential agonistic conflict, individuals must estimate the likelihood of victory or defeat, and the costs likely to be incurred therein. In calculating such probabilities, actors have to assess many attributes that the parties bring to the interaction. Because these attributes are diverse, decision-making can be facilitated by compiling assessments of the relevant features into a single representation, a summary that can then readily be consulted. Physical size and strength have been fundamental determinants of the outcomes of violent conflicts throughout our species’ evolutionary history, a pattern recapitulated in experience during development. Physical size and strength thus provide readily available dimensions for such a summary representation. Based on this logic, Fessler, Holbrook, and Snyder [1] argued that the mind employs the conceptual dimensions of physical size and strength to summarize multiple factors that contribute to the likely outcome of a conflict: in essence, the decision-maker builds a mental image of the opponent, adjusting the physical size and strength depicted therein as each item of relevant information about the opponent or the self is evaluated. Consonant with this perspective, knowing that another individual is armed increases participants’ estimations of the target’s size and muscularity [1]; conversely, similar estimations are decreased by the presence of the participant’s allies, who could assist in a fight [2]. Likewise, knowing that the leader of a violent coalition has either been killed, or, alternately, experienced military successes, leads participants to respectively decrease or increase their estimations of the size and strength of a representative member of that coalition [3]. Lastly, directly tapping issues of physical ability, men’s own strength is inversely related to their estimations of an antagonist’s physical formidability [4].

While the above work focuses on decision making in agonistic situations, complementary evidence has been presented by investigators exploring the use of conceptual metaphors in reasoning about social hierarchy. Specifically, drawing on observations that the vertical dimension is used to represent hierarchical relationships (e.g., [5], [6]), Yap, Mason, and Ames [7] found that manipulating participants’ sense of power shaped their estimates of another individual’s size and weight, such that participants made to feel powerful underestimated these dimensions, while participants made to feel powerless overestimated them. Similarly, Duguid and Goncalo [8] demonstrated that participants made to feel powerful overestimated their own height and underestimated the height of another person (a pattern consistent with the qualia associated with emotions active during dominance interactions – e.g., [9]). Thus, conceptualizations of physical size and strength appear to be deployed in reasoning about both relative formidability in particular and social power in general.

At the most elementary level, in the absence of other factors, the actor’s ability to respond effectively to threatening situations hinges on physical attributes. Strength is not the only consideration in this regard, as even strong individuals may be handicapped by burdens or injuries. If the actor’s representation of a prospective antagonist summarizes the assets and liabilities that the two parties bring to a conflict, such a conceptualization should therefore be affected by any temporary incapacitation that the actor suffers at the time of assessment. Hence, if size and strength constitute the dimensions of a summary representation of relative formidability, then being incapacitated should lead actors to increase their conceptualizations of a given foe in these regards. This prediction can also be framed in the language of social hierarchy: to the extent that one’s social power is conceptualized in terms of bodily attributes, such that possession of power affects perceptions of the size of others relative to oneself, then physical incapacitation – which reduces social power – should lead actors to perceive a target individual as larger. In our successful tests of this prediction, we experimentally manipulated physical incapacitation in two studies. Because men engage in far more violence than do women [10], and, correspondingly, physical capabilities are intimately linked to men’s propensities for aggression [11]–[15], we expected the predicted effect to be most marked in men. Accordingly, given the exploratory nature of this project, we limited our investigation to men. Similarly, because our hypothesis specifically concerns agonistic interactions, in order to present the clearest test of the hypothesis, we employed angry male faces as the targets of participants’ assessments, thereby implicitly framing any potential interaction as likely to be agonistic.

## Ethics Statement

Both studies reported here were examined and approved by the University of California, Los Angeles Institutional Review Board. Following the exact protocol approved by the Institutional Review Board, in each study, participants were first presented with a written information sheet describing the study procedures, any potential risks or discomforts, the identity and contact information of the first author, and compensation; participants then indicated their consent to participate. The protocol approved by the Institutional Review Board dictated that, in order to ensure participant anonymity, consent was given orally, rather in writing, with anonymous identification numbers being assigned to each participant in order to document this procedure.

## Study 1

### Participants

Fifty-one male undergraduates at the University of California, Los Angeles were recruited to participate in a study, advertised as concerning links between physical disability and visual perception, in exchange for $8. Data were screened prior to analysis for overt suspicion regarding the hypothesis or frivolous responses. There were no such concerns, but technical problems led to incomplete data for 5 participants, leaving a final sample of 46 men, with a mean age of 22.9 years (*SD = *6.69). The ethnicity of the sample was 40.4% White, 25.5% East Asian, 10.6% Latino, 8.5% Black, 4.3% South Asian, 4.3% Middle Eastern, and 6.4% other or mixed ethnicities.

### Materials and Methods

Upon arriving at the laboratory, participants’ heights were measured. Then, in a within-subjects counterbalanced design, participants were asked to experience both simulated near-total quadriplegia (the impaired condition) and minor nerve damage to the fingers (the control condition). In the impaired condition, participants were strapped tightly to a wooden chair using six nylon webbing straps attached just above the ankles, on the lower forearms, and at the elbows; the chair itself was anchored in place using 136 kilograms of weights. Care was taken not to cause pain or impair circulation, and the hands were free to move at the wrist. A numeric response pad was positioned adjacent to the chair, within the participant’s reach. After being fastened to the chair, participants were asked to attempt to move; the straps were then tightened as needed. This step ensured both that participants were truly incapacitated and that they were cognizant of the extent of their incapacitation. In the control condition, participants were seated in the same chair and used the same response pad, but were unrestrained; consonant with the framing of the study as concerning physical disabilities, small metal caps were attached to the fingertips of the dominant hand, ostensibly to simulate minor peripheral nerve damage. Once the participant had been readied, the research assistant moved behind a privacy screen, remaining in the room.

**Figure 1:**
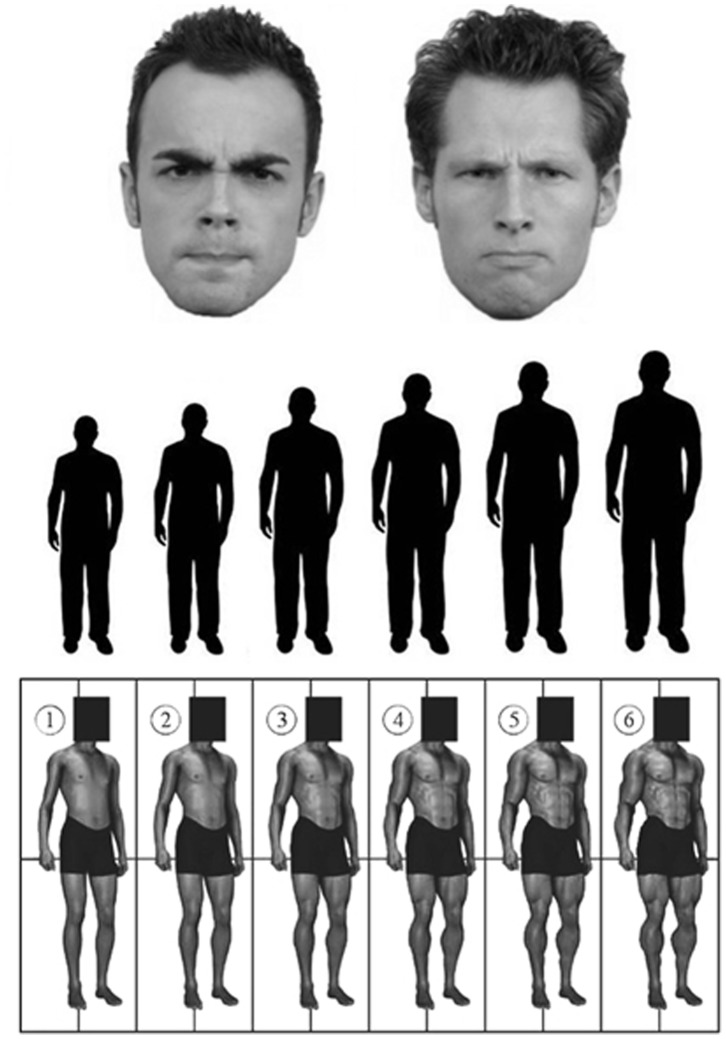
In two studies using within-subjects, counterbalanced designs, participants estimated the height, size, and muscularity of male targets presented as angry faces (taken from the Radboud Faces Database, [16]) while impaired or unimpaired.

Participants completed parallel tasks in each condition, beginning with one of two versions of a computerized survey, presented in counterbalanced order across conditions. The surveys were projected on a 113 cm by 71 cm monitor positioned 168 cm away, directly in front of the chair. The surveys included relative formidability measures interspersed among filler visual judgment measures (e.g., participants were asked to estimate how many jellybeans or seashells were in a partially occluded container) intended to reduce demand characteristics. In the formidability measure, participants were shown images of angry White male faces (one per survey) and asked to estimate the individual’s i) height in feet and inches, ii) overall size using a 6-point array, and iii) muscularity using a 6-point array (see Figure 1). The angry face stimuli were cropped and grayscale-converted images modified from the Radboud Faces Database [16]; models posing for this database consented to have their images used in scientific research and disseminated in scientific publications (see http://www.socsci.ru.nl:8180/RaFD2/RaFD?p=faq; accessed March 3, 2013).

The self-height estimation task was administered next, while the participant was still seated in the same location. The privacy screen, chair, and an 8-foot (2.5-meter) stadiometer were located such that participants could see only the unmarked side of the monochromatic stadiometer, located 380 cm away in front of a blank white wall; the stadiometer’s units of measure were visible only to the research assistant. While remaining behind the privacy screen, using a laser pointer, and beginning at the base of the stadiometer, the research assistant moved a point of light upwards at a rate of 10 centimeters per second. Participants were instructed to say “Stop” at the precise moment when the laser dot reached the level that they believed matched their standing height.

Individuals differ in a variety of traits affecting their ability to win physical conflicts. Such differences are plausibly reflected in people’s subjective concerns regarding the possibility of being criminally victimized [17]. Correspondingly, such concerns have previously been linked with estimates of a potential antagonist’s size and strength [2]. Accordingly, once the two physical impairment conditions had been completed, participants were seated in another chair and answered demographic questions and a five-item fear of crime measure (α = .92) (modified from the British Fear of Local Crime Survey – [18]) examining their level of concern about five types of victimization (e.g., mugging, theft) using a 7-point Likert scale (1 = *Not worried at all*, 7 = *Very worried*). As an exploratory measure of the contribution of social power to estimations of the targets’ physical formidability and of participants’ own height, self-perceived status was assessed using the MacArthur Scale of Subjective Social Status [19]. This scale depicts an image of a ladder representing the spectrum of socioeconomic statuses in the United States: participants select the rung that reflects their overall status relative to others.

Finally, participants were questioned for suspicion about the purpose of the study. Although several participants speculated that the study involved visual bias related to incapacitation, none evinced suspicion that such bias concerned inflating the physical attributes of the target males. Participants were then thanked, paid, and debriefed.

### Results

Preliminary MANOVAs revealed no significant effects of order of condition or survey version on estimations of the height, size, or muscularity of the angry male targets, or on estimates of participant’s own height.

#### Envisioned height, size, and muscularity of the angry males

As predicted, angry male targets were estimated to be taller in the impaired condition (*M* = 70.92 inches, *SD* = 2.55) than in the control condition (*M* = 69.98 inches, *SD* = 2.15), *F*(1, 45) = 6.02, *p*<.02, *η* ^2^_p = _.12. The target was also estimated to be larger using the silhouette array in the impaired condition (*M* = 4.13, *SD* = .75) than in the control condition (*M* = 3.63, *SD* = .90), *F*(1, 45) = 11.37, *p*<.01, *η* ^2^_p = _.20. The target was estimated to be slightly more muscular in the impaired condition (*M* = 2.28, *SD* = .86) than in the control condition (*M* = 2.09, *SD* = .96), but this difference was not significant, *p* = .21.

To assess whether fear of crime positively correlated with perceptions of the targets as more formidable in the impaired condition, difference scores were calculated subtracting the height, size, and muscularity of the target estimations made while in the control condition from those made while restrained. Although all correlations were in the predicted positive direction, there were no significant correlations between subjective fear of crime and difference scores for height or size, *p*s >.4. However, there was a nonsignificant trend linking fear of crime with muscularity estimates *r*(45) = .27, *p*<.08. There were no significant correlations between perceived social status and the difference scores for estimated height, size, or muscularity, *p*s >.3.

#### Perceptions of self-height

Consistent with predictions, participants assessed their own height as shorter in the impaired condition (*M* = 64.13 inches, *SD* = 6.17) than in the control condition (*M* = 67.59 inches, *SD* = 6.83). A repeated measures ANOVA confirmed that the effect of impairment condition on estimations of self-height was significant, *F*(1, 44) = 11.56, *p = *.001, *η* ^2^_p = _.21.

To assess whether fear of crime correlated with perceptions of oneself as shorter when restrained, difference scores were calculated subtracting the self-height estimation made when restrained from that made when unrestrained. As predicted, subjective fear of crime predicted estimations of one’s own height as shorter when restrained versus unrestrained, *r*(45) = .35, *p* = .02.

In a marginal trend, self-perceived social status was positively correlated with the difference between estimations of own height when restrained versus unrestrained, *r*(45) = .27, *p*<.08. A follow-up test indicated that this was driven by a correlation between status and own height in the unrestrained condition, *r*(45) = .25, *p*<.1; there was no correlation in the impaired condition, *p*>.65. The positive trend in the control condition is consistent with prior findings that enhanced perceptions of social power inflate estimates of own height [8].

### Discussion

Consonant with our thesis that physical incapacitation should alter the conceptualized relative formidability of a potential antagonist, we found that restraining U.S. male undergraduate students’ arms and legs led them to envision an unfamiliar angry man as larger. Similarly, in keeping with prior work showing that relative power affects assessments of one’s own size, this manipulation substantially decreased participants’ visual assessments of their own height. However, despite these positive results, the predicted influence of physical restraints on participants’ conceptualization of a prospective foe as more muscular was not significant.

Importantly, this laboratory study was notably divorced from the sorts of everyday contexts in which the psychology at issue could be expected to operate. Having one’s arms and legs bound to an immobile chair is an extreme instantiation of the sort of incapacitation that able-bodied people would normally experience periodically. Correspondingly, both the oddness of the experience and the anxiety that may have attended it could have affected participants’ responses in unknown ways. To address these limitations, we ran a second study, conducted in public areas, in which participants were asked to engage in a simple and relatively familiar balance task in order to induce the experience of partial incapacitation.

## Study 2

Postural instability can result from fatigue [20], injury [20], illness e.g., [21], intoxication, or merely loose or uneven terrain. Regardless of cause, in a physical confrontation, postural instability constitutes a handicap. Accordingly, if relative formidability is conceptualized in terms of a potential antagonist’s size and strength, then the experience of temporary postural instability should lead individuals to envision a foe as physically larger and more muscular. This experience is readily induced by having participants stand on an unstable platform. Moreover, in contrast to the experience of being physically restrained, standing on an unstable platform is a familiar challenge for members of the population studied here – indeed, precisely this task is routinely used in both athletic training and medical rehabilitation. A balance board is a wooden disk resting atop a domed protrusion, creating a teetering, rotating surface on which fitness enthusiasts and patients attempt to stand. In Southern California, balance boards are found in many gyms and rehabilitation facilities, and inexpensive home versions are readily available, hence we expected them to be familiar to many participants in our fitness-minded community. Employing the same key dependent measures used in Study 1, we examined how the mundane task of standing on a balance board affected participants’ conceptualizations of a potential antagonist. By virtue of the commonplace nature of balance boards in our area, we anticipated that some participants would be adept at using them. Because our goal was to induce an experience of postural instability sufficient to entail substantial disadvantage in a violent conflict, as a manipulation check we collected participants’ subjective assessments of the difficulty of standing on the balance board.

### Participants

Ninety-eight adult men were recruited in public areas on or near the University of California, Los Angeles campus in exchange for $3. The study was presented as testing links between motor skills and visual skills. Data were screened prior to analysis for overt suspicion, frivolous or incomplete responses, and perception of the balance task as unchallenging. One individual evinced suspicion, one gave frivolous responses (estimating the male target to be under 4 feet tall), six provided incomplete data, and 45 individuals rated the balance board task as unchallenging, leaving a final sample of 45 men, with a mean age of 24.7 years (*SD = *8.02). The ethnicity of the sample was 53.3% White, 22.2% Asian, 8.9% Latino, 4.4% Black, 2.2% Middle Eastern, and 8.9% other or mixed ethnicities.

### Materials and Methods

Participants were recruited while walking alone, as prior research [2] has found that the presence of allies diminishes representations of the formidability of threatening male targets. For safety reasons, and to avoid potential confounds, men with motor impairments or injuries were excluded. Upon recruitment, participants were escorted away from any groups of passersby. In a within-subjects counterbalanced design, participants completed two parallel survey packets, presented on clipboards. In the experimental condition, participants filled out the survey while using a balance board; in the control condition, participants did so while standing on the ground. Participants were instructed to try to prevent the sides of the board from touching the ground while balancing.

The visual filler and formidability items were similar to those used in Study 1. Participants were shown images of the same angry male faces used previously, and asked (in fixed order) to estimate the individual’s height, overall size, and muscularity using the same measures as in Study 1. The two versions of the survey were presented in counterbalanced order.

After completing both surveys, participants answered demographic questions and the fear of crime measure (α = .91) used in Study 1. Self-perceived social power was not assessed, as the design included neither estimations of personal height nor a manipulation likely to moderate social power. As a manipulation check, the subjective difficulty of balancing on the board was reported using a 9-point scale (1 = *Extremely easy*; 9 = *Extremely difficult*). Finally, participants were questioned for suspicion. Although several speculated that the study might involve visual bias related to being off-balance, only one evinced suspicion that such bias concerned inflating the physical attributes of the pictured angry males; this participant’s responses were removed prior to analysis.

### Results

#### Manipulation check

Exactly half of the sample (50%) rated the balancing task as less than minimally difficult (i.e., at or below the midpoint of the 9-point scale). As individuals responding in this fashion likely did not undergo the experience of extensive postural instability that our manipulation was intended to induce, our principal analyses exclude these participants, encompassing only those participants who rated the balancing task as at least minimally difficult. Following presentation of our principal analyses, we also present exploratory analyses of the complete sample.

Preliminary MANOVAs revealed no significant main effect of order of condition on estimations of the height, size, or muscularity of the angry male targets, *p*>.3. However, there was a main effect of survey sequence (i.e., which packet was completed first), *p*<.04, with significant effects of survey sequence on estimates of height and muscularity, *p*s <.04. The survey sequence was therefore controlled for in subsequent analyses of height and muscularity estimates.

#### Envisioned height, size, and muscularity of the angry males

As predicted, the target was estimated to be taller in the balance board condition (*M* = 70.34 inches, *SD* = 2.78) than in the control condition (*M* = 69.29 inches, *SD* = 2.24), *F*(1, 43) = 4.16, *p*<.05, *η* ^2^_p = _.09. Likewise, the target was estimated to be larger using the silhouette array in the balance board condition (*M* = 4.07, *SD* = 1.12) than in the control condition (*M* = 3.63, *SD* = 1.07), *F*(1, 44) = 4.33, *p*<.05, *η* ^2^_p = _.09. Lastly, the target was also estimated to be more muscular in the balance board condition (*M* = 2.98, *SD* = 1.22) than in the control condition (*M* = 2.71, *SD* = 1.06), *F*(1, 43) = 4.81, *p*<.04, *η* ^2^_p = _.10. Difference scores between estimates of height, size, and muscularity made between conditions were calculated, then correlated with fear of crime ratings; fear of crime did not correlate with any of these scores, *p*s >.18.

#### Effects of balance difficulty

To explore the influence of task difficulty on formidability estimation, we repeated the above tests on the half of the sample composed of participants who did not rate the balance task as minimally difficult or above. Consistent with predictions, there were no significant effects of condition for any of the three bodily estimations, *p*s >.5. To further assess whether task difficulty moderated the effect of condition, examining the entire sample (*N* = 90), we correlated the difficulty ratings with the difference scores for estimated height, size, and muscularity between conditions. Unexpectedly, task difficulty did not correlate with any of these difference scores, *p*s >.5. This evident absence of moderation contrasts with the striking lack of effect of condition observed among the portion of the sample that did not rate the balance task as minimally difficult or above. It is possible that, below this threshold, the finer gradations in our self-reported difficulty measure did not closely track the extent to which the manipulation influenced perceived relative formidability. Seeking to more precisely identify the floor in self-reported difficulty below which the manipulation had no effect on perceived relative formidability, we next assessed the effect of condition on all participants whose difficulty scores were greater than one standard deviation below the mean, a subsample (*N* = 65) composed of those who rated the task as a 4 or above on our 9-point scale. Indeed, in this enlarged sample, the target was estimated to be taller in the balance board condition (*M* = 70.25 inches, *SD* = 2.58) than in the control condition (*M* = 69.42 inches, *SD* = 2.32), *F*(1, 64) = 4.83, *p*<.04, *η* ^2^_p = _.07. Likewise, in a nonsignificant trend, the target was estimated to be larger in the balance board condition (*M* = 3.89, *SD* = 1.10) than in the control condition (*M* = 3.68, *SD* = 1.05), *F*(1, 64) = 3.21, *p*<.08, *η* ^2^_p = _.05. However, although the angry male targets were also estimated to be slightly more muscular in the balance board condition (*M* = 2.86, *SD* = 1.20) than in the control condition (*M* = 2.75, *SD* = 1.02), this difference was not significant, *p*>.4. In sum, the overall pattern suggests that the null effects of condition observed in the half of the sample who rated the balance task as unchallenging were driven by the participants who rated the task as relatively easy, with modest evidence of an influence of the manipulation among those who rated the task as less easy, and, as expected, the most pronounced evidence of an influence of the manipulation among those participants who reported the task to be difficult.

### Discussion

Study 2 replicated the key results of Study 1 using a more mundane manipulation of physical incapacitation, conducted in a public setting rather than a laboratory, and absent the overt priming of physical infirmity involved in the framing of Study 1. For those participants who found standing on a balance board to be challenging, a potential antagonist was estimated to be more physically formidable when the participant was handicapped by postural instability than when such estimates were provided with the benefit of firm footing. Similar, albeit reduced, effects were evident among participants who found the task to be less challenging; as expected, these effects were absent among those who found the task to be relatively easy (and thus presumably experienced minimal instability). Our measures of the estimated height and overall size of a potential antagonist revealed the predicted effects of incapacitation in both studies; additionally, in Study 2, we also found a significant effect of incapacitation on estimates of the muscularity of the target, a pattern discernable as a nonsignificant trend in Study 1. Fear of crime again did not correlate with estimates of target formidability, suggesting that this self-reported subjective attribute may have limited applicability in such investigations.

### Conclusions

In two studies, using very different means of inducing temporary physical incapacitation, we found that this state led participants to increase their conceptualizations of the physical formidability of potential antagonists; correspondingly, Study 1 also revealed that incapacitation led participants to conceptualize themselves as smaller.

Our results are largely redundant across differing manipulations. Nevertheless, our findings are subject to sampling limitations, as we studied only young men in and around one Southern Californian university. At the least, it will be important in future studies to examine the effects of incapacitation among women; ideally, future studies will also expand beyond Western university contexts. Likewise, because we sought to maximize the likelihood that participants would perceive the individual being evaluated as a potential antagonist, we employed only angry male faces as target stimuli; in the future, it will be important to examine the full extent of the phenomena explored here by varying both the emotions expressed by the target and the target’s sex.

Our investigations focus on how the mind summarizes assessments of relative formidability using the dimensions of size and muscularity. Although we are concerned with cognitive representations in the mind of the perceiver, size and muscularity are also morphological attributes of the target individual, features that are subject to inspection. We therefore presented participants with intentionally underspecified stimuli so that, rather than reflecting their perceptual accuracy or attention to the details of the stimuli, their estimations of the bodily characteristics of the target individual would instead reveal their representations of relative formidability. Indeed, ceteris paribus, we expect perceptual accuracy to be unaffected by factors affecting relative formidability, as, regardless of whether the ensuing decision is to fight or flee, the effectiveness of subsequent action hinges on such accuracy, hence natural selection can be expected to disfavor diminished accuracy [2]. However, perceptual accuracy is contingent on attention, and attention is a finite cognitive resource. The latter consideration suggests that attention to the determinants of a potential antagonist’s relative formidability, including his actual size and muscularity, should be a function of the need for accuracy in the given situation. Because the costs of failing to detect substantial formidability in an antagonist are greater for individuals who are less formidable than they are for individuals who are more formidable, the former should devote more attention to cues of relative formidability than the latter [22]. Correspondingly, shorter men are more sensitive than taller men to facial and vocal cues of dominance in a male target [22], and priming men with the experience of defeat similarly up-regulates sensitivity in this regard [23]. In the future, it will be important to explore the interactions between the determinants of relative formidability, the allocation of attention to these determinants, and internal representations of relative formidability.

Our findings are potentially explicable in terms of both the narrower view that the conceptualized size and strength of an antagonist summarizes relative formidability and the broader view that size is used to represent social power. Study 1, in which participants were physically restrained, created both the experience of the tactical liability of incapacitation and, by virtue of the fact that the most elementary form of social power involves physical coercion, the experience of powerlessness. With regard to Study 2, although we have framed the consequences of standing on an unstable balance board in terms of the disadvantages of postural instability, it is possible that this experience was processed not in terms of a temporary handicap, but rather as feedback regarding athletic competence. Arguably, for young men in a fitness-oriented community, athletic competence is a determinant not only of relative formidability, but also of social status – and thus power. However, were this the central factor, we would expect to have found an order effect in Study 2: the effects of feedback regarding social status should not dissipate within moments, hence participants who completed the experimental condition before the control condition should have shown lower effects of the manipulation than did participants who experienced the reverse order of conditions. This was not the case, hence the observed effects likely primarily reflect assessments of relative formidability, not relative social power. Lastly, the representations-of-formidability view predicts that, in addition to increasing the envisioned physical height and overall body size of a potential antagonist, incapacitation should also increase the envisioned muscularity of the target individual; the representations-of-power view is silent on this issue. We found positive support for this pattern in Study 2, and a corresponding trend in Study 1.

The two theoretical frameworks outlined above, though highlighting distinct functional computations regarding, respectively, combat and social position, are rendered complementary by an evolutionary approach to the psychology of power. In nonhuman animals, power stems from dominance – the ability to achieve goals through force or the threat thereof. However, in many human groups, power stems not from dominance, but from either prestige or formal office: rather than using physical force to achieve their goals, people rely on the social status awarded them by others [24],[25]. Whether through social capital or formal authority, social position thus translates into power without the use of violence. Viewed against the backdrop of primate sociality, these types of power are evolutionary innovations. Natural selection acts primarily through the modification of existing traits rather than the creation of new mechanisms [26], hence evolutionarily more recent features often reflect their earlier origins. Moreover, when the adaptive challenge addressed by the ancestral trait continues to exist, relevant features of that trait are preserved in the derived trait. Both considerations apply to the question of power, as i) both prestige-based and office-based power evolutionarily postdate dominance-based power, and ii) the advent of prestige-based and office-based power has not eliminated dominance-based power. We can therefore expect the psychology of prestige-based and office-based power to reflect the psychology of dominance-based power (see [27], [28]). Accordingly, if the ability to win a violent conflict is represented using the conceptualized size of the opponent, then the same should be true of representations of relative power – whether or not that power derives from the threat of violence. In sum, size metaphors are likely brought to bear in reasoning about both combat and status because these domains are deeply connected in the evolved mind, which thriftily re-uses mechanisms whenever possible. The corpus of results to which the present findings add suggests that this is indeed the case, providing grounds for future explorations of the connections between bodily experience, conceptions of relative bodily size, and the dynamics of social conflict and hierarchy.
